# Development and Bioavailability Assessment of an Estriol-Containing Vaginal Hydrogel

**DOI:** 10.3390/gels10120823

**Published:** 2024-12-13

**Authors:** Peter Takacs, Barbara Kozma, Dávid Rátonyi, Bence Kozma, Kiss-Szikszai Attila, Ferenc Fenyvesi, Attila G. Sipos

**Affiliations:** 1 Division of Female Pelvic Medicine and Reconstructive Surgery, Department of Obstetrics and Gynecology, Eastern Virginia Medical School, 825 Fairfax Avenue, Suite 526, Norfolk, VA 23507-2007, USA; takacsp@evms.edu; 2 Department of Obstetrics and Gynecology, Faculty of Medicine, University of Debrecen, Nagyerdei krt. 98, 4032 Debrecen, Hungary; kozma.barbara@med.unideb.hu (B.K.); ratonyi.david@med.unideb.hu (D.R.); bence.kozma@med.unideb.hu (B.K.); 3 Doctoral School of Nutrition and Food Sciences, University of Debrecen, Egyetem tér 1, 4032 Debrecen, Hungary; 4 Department of Organic Chemistry, Faculty of Science and Technology, University of Debrecen, Egyetem tér 1, 4032 Debrecen, Hungary; kiss.attila@science.unideb.hu; 5 Department of Molecular and Nanopharmaceutics, Faculty of Pharmacy, University of Debrecen, Rugó utca, 4002 Debrecen, Hungary; fenyvesi.ferenc@pharm.unideb.hu

**Keywords:** genitourinary syndrome of menopause (GSM), estriol-containing vaginal gel, hydroxyethyl cellulose (HEC), hydroxypropyl-β-cyclodextrin (HPBCD), estriol

## Abstract

Genitourinary syndrome of menopause (GSM) affects a significant percentage of postmenopausal women and manifests as vaginal dryness, irritation, and urinary discomfort, typically treated with vaginal estrogens. Hydrogels are preferred over creams due to their superior comfort and mucoadhesive properties. This study introduces a novel vaginal gel formulation containing hydroxyethyl cellulose (HEC) and estriol-hydroxypropyl-β-cyclodextrin complex (E3-HPBCD) for the treatment of GSM. The estriol (E3) release profile of the gel was evaluated using a Franz diffusion cell system, and its permeability was tested on reconstructed human vaginal epithelium. Biocompatibility was assessed using (3-[4,5-dimethylthiazol-2-yl]-2,5-diphenyltetrazolium bromide) (MTT), lactate dehydrogenase (LDH) assays, and real-time cell analysis (RTCA) on human skin keratinocyte (HaCaT) cells, which showed increased cell viability and no obvious cytotoxicity. The results indicated that efficient E3 release and satisfactory epithelial permeability with HPBCD provide the bioavailability of E3. These results suggest the potential of the gel as a biocompatible and effective alternative for the treatment of GSM. Further studies are required to assess the long-term safety and clinical efficacy.

## 1. Introduction

Genitourinary syndrome of menopause (GSM), formerly known by terms such as vaginal atrophy, vulvovaginal atrophy, urogenital atrophy, or atrophic vaginitis, encompasses a spectrum of symptoms and clinical signs resulting from estrogen deficiency, which affects the lower genital tract, urinary tract, and external genitalia [1]. Multiple studies estimate the prevalence of GSM to be at least 40–50% [2,3,4]. The significance of GSM extends beyond its high prevalence. The condition presents with symptoms such as vaginal dryness, burning, and irritation. Sexual symptoms include reduced lubrication, discomfort or pain, and sexual dysfunction, as well as urinary symptoms like urgency, dysuria, and recurrent urinary tract infections. These manifestations have a profound negative impact on the sexual health and overall quality of life of postmenopausal women [5].

The traditional primary approach to treating GSM has been vaginal estrogen therapy. International guidelines recommend using lubricants and moisturizers as first-line treatments. In more severe cases where these interventions are insufficient, vaginal estrogen therapy may be considered. According to the American College of Obstetricians and Gynecologists (ACOG) and the North American Menopause Society (NAMS), systemic estrogen therapy is not recommended for GSM; instead, topical vaginal estrogen is preferred [6,7]. Several vaginal estrogen formulations are available, including those containing conjugated estrogens, estradiol (E2), or estriol (E3). E3, the end product of estrogen metabolism, cannot be converted back to estrone or estradiol, while estrone and estradiol can be metabolized interchangeably. E3 has a much lower affinity for estrogen receptors, about ten times lower than E2, and a shorter duration of action in the cell nucleus. Due to its low binding to plasma proteins and rapid clearance from the body, E3 is considered a short-acting estrogen [8]. According to systematic reviews, intravaginal E3 is cost-effective at low doses and can be used safely in women with a history of breast cancer or thromboembolic disease, with minimal side effects [9,10,11]. Another systematic review concluded that the use of vaginal E3 does not induce endometrial proliferation, a finding confirmed by hysteroscopic sampling and histological analysis [12].

The effectiveness of vaginal estrogen cream for treating vaginal atrophy is well-established; however, many women tend to avoid creamy vaginal applications for several reasons related to comfort. Creamy formulations can often feel messy and sticky, leading to discomfort, especially during daily activities. Ointments are generally semi-solid, smooth, and spreadable preparations intended for use on the skin or mucous membranes. While they appear homogeneous, their formulation is distinct from the structure of living tissue [13]. Hydrogels generally offer a more comfortable application experience compared to creamy vaginal applications. In our previous study, 94% of participants reported that the hydrogel was comfortable to use, while 85% found its consistency to be highly satisfactory [14].

Hydrogels are hydrophilic materials characterized by a three-dimensional crosslinked molecular structure that allows them to absorb large amounts of water without dissolving in aqueous or physiological environments [15]. Hydrogels have physical properties similar to living tissues due to their high water content, elastic consistency, and low interfacial tension with biological fluids and water [16]. Hydrogels are commonly used in medicine. Among many other possibilities, a recent study revealed that a chitosan-based hydrogel is a viable option for the vaginal administration of progesterone [17]. Inert and active hydrogels also play a significant role in burn first aid and the treatment of infected wounds [18,19]. While several hydrogels for vaginal use are available over the counter as lubricants or moisturizers, there have been very few clinical trials conducted to demonstrate their safety and effectiveness [14,20].

Cyclodextrins (CDs) are valuable formulation agents that enhance the solubility of drugs in aqueous solutions, thereby improving the delivery of various medicinal compounds to biological systems. Effective delivery systems are crucial, as many drugs would not be viable for development without them [21]. The entire CD molecule is water soluble. However, the interior of its cup-shaped structure is relatively nonpolar, forming a hydrophobic micro-environment [22]. These characteristics contribute to CDs’ water solubility and capacity to encapsulate hydrophobic substances within their cavities [23]. 2-hydroxypropyl-β-cyclodextrin (HPBCD) is the most versatile excipient among cyclic oligosaccharides, suitable for use in oral, rectal, dermal, ocular, and parenteral formulations [24]. The list of approved and marketed medicines containing CDs as an excipient is constantly growing. More than 120 active ingredients are already on the market in drug formulations containing CDs as excipients [25]. Our research group has previously tested a zinc-containing vaginal gel with excellent biocompatibility and clinical properties in clinical trials, which has formed the basis of the E3-HPBCD complex gel [14,26]. E3 is a lipophilic molecule and cannot be dissolved and distributed homogenously in a hydrogel without suitable excipients. HPBCD is known to form inclusion complexes with estrogens; therefore, it was selected for the formulation of the E3-containing hydrogel [27]. The primary aims of this study were to assess the in vitro biocompatibility of a vaginal gel containing E3 and zinc, evaluate the gel’s E3-release profile in the presence of zinc, and investigate the transepithelial permeability of E3 in a reconstituted human vaginal epithelium model. We hypothesized that E3 would be efficiently released from the zinc-containing gel and effectively permeate the vaginal epithelium.

## 2. Results and Discussion

### 2.1. Results

#### 2.1.1. In Vitro Release and Membrane Permeation of E3

**Figure 1:**
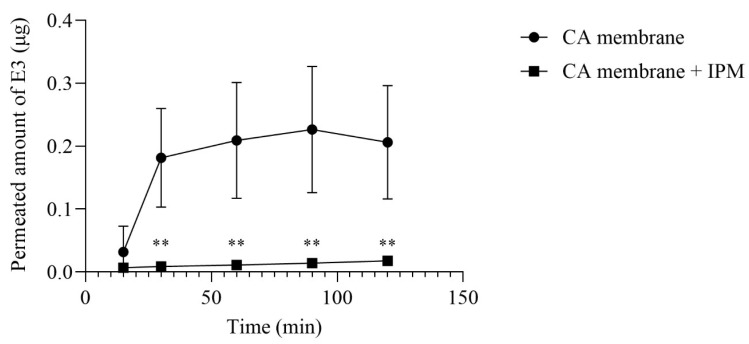
E3 permeation through non-impregnated and IPM-impregnated cellulose-acetate (CA) membranes in the function of time. Values were expressed as mean ± S.D., n = 4–6. ** represents *p* < 0.01 compared to the untreated control.

Figure 1 shows that E3 is liberated from the E3 gel and permeates the cellulose-acetate membranes. Impregnation of membranes with IPM significantly decreased the permeating amount of E3 (*p* < 0.01). 

#### 2.1.2. Biocompatibility Tests on HaCaT Cells

**Figure 2:**
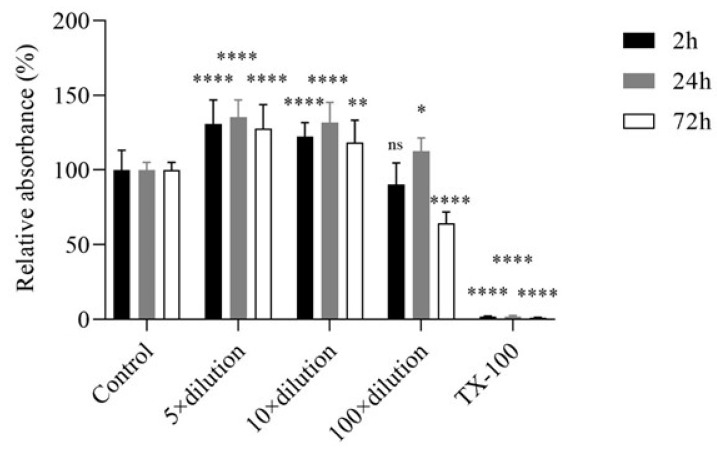
The effect of E3 gel on HaCaT cells after 2, 24, and 72 h of incubation in a complete medium was measured by an MTT test. E3 gel was diluted by the complete medium 5, 10, and 100 times before the experiment for applicability. Values were expressed as the mean ± S.D., n = 4–6. *, **, and **** represent *p* < 0.05, *p* < 0.01, and *p* < 0.0001 compared to the untreated control. ns: not significant.

The biocompatibility of E3 gel was tested on HaCaT cells first. Cells were treated with 5-, 10-, and 100-times dilutions of E3 gel in complete medium, and after 2, 24, and 72 h of incubation, the cell viability was determined by an MTT assay. As Figure 2 shows, the dilutions of E3 gel significantly increased the formation of the formazan dye in HaCaT cells and the relative absorbance of the samples compared to the untreated control. TX-100 completely abolished cell viability.

**Figure 3:**
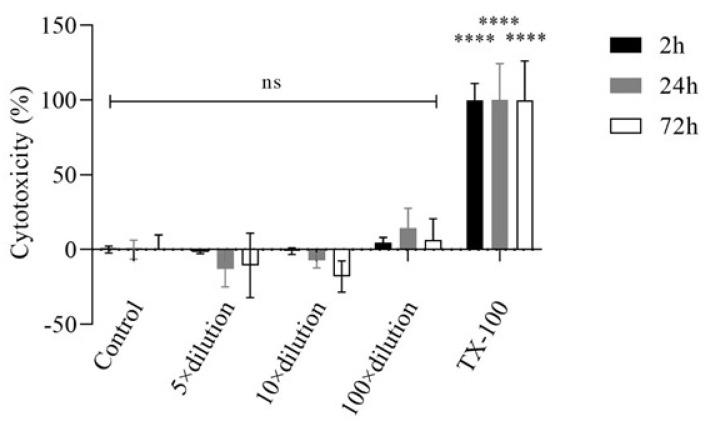
The effect of E3 gel on HaCaT cells after 2, 24, and 72 h of incubation in a medium containing 1% FBS, measured by the LDH test. Values were expressed as mean ± S.D., n = 6. **** represents *p* < 0.0001 compared to the untreated control. ns: not significant.

To further study the effect of E3 gel on HaCat cells, the cytotoxic effect of the gel was measured by LDH release. The experiments were carried out in a medium containing 1% FBS to avoid the high background of the serum. Figure 3 shows that the treatment of HaCaT cells with the dilutions of the E3 gel did not significantly increase the measured cytotoxicity of the gel. However, the positive control TX-100 caused complete cytotoxicity.

#### 2.1.3. Real-Time Cell Analysis (RTCA)

**Figure 4:**
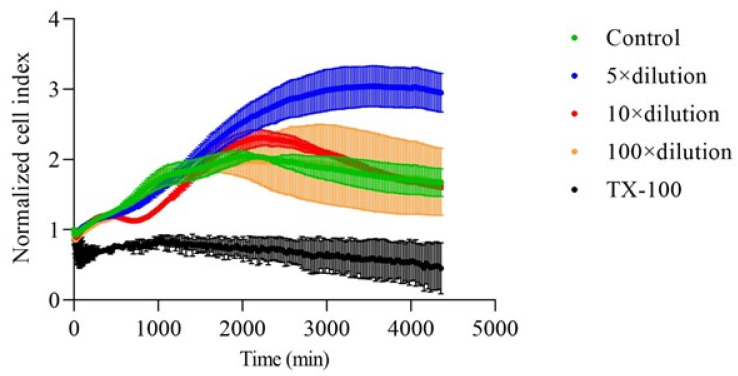
The effect of E3 gel on the proliferation of HaCaT cells was measured by the RTCA method. E3-gel-treated cells were diluted in complete medium 5, 10, and 100 times and the normalized cell index was registered for 72 h after treatment. We found that 5 × dilution of the gel increased significantly (*p* < 0.0001), while TX-100 decreased significantly (*p* < 0.0001) in the normalized cell index compared to the untreated control.

The effect of E3 gel on the proliferation of HaCaT cells was tested using the real-time cell analysis method. After treatment, the impedance was recorded every 25 min, and the instrument software calculated the normalized cell index. As Figure 4 shows, the treatment of cells with the 5-times dilution of the gel significantly increased the maximum of the curve of the normalized cell index, which shows a higher cell number or stronger attachment of the cells on the electrodes. Treatment with 10- and 100-times dilutions did not cause significant alterations, and TX-100 treatment significantly decreased the cell index.

#### 2.1.4. Biocompatibility Tests on Reconstructed Human Vaginal Epithelium

**Figure 5:**
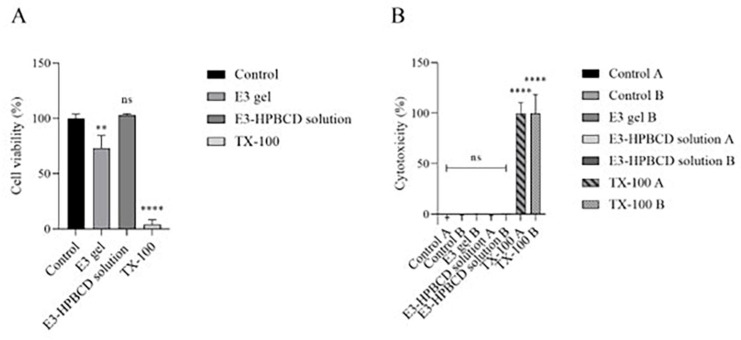
Results of the biocompatibility on reconstructed human vaginal epithelium measured by MTT (**A**) and LDH tests (**B**). Human vaginal epithelial cells were grown on porous inserts and treated with E3 gel or E3-HPBCD solution from the apical side. Samples for the LDH-release measurements were collected both from the apical side of the inserts and the basal side (indicated by “A” and “B” in the figure legends, respectively). Values are presented as means ± S.D., n = 3. ** and **** represent *p* < 0.01 and *p* < 0.0001, respectively, compared to the untreated control, ns: not significant.

The biocompatibility of E3 gel and its main components was tested on reconstructed human vaginal epithelium. Cell layers grown on porous inserts were treated with the undiluted gel, and the solution of its main components and their effects were tested by MTT and LDH tests. Figure 5A shows the results of the MTT assay. The relative absorbance of the formazan dye, indicating cell viability, did not decrease below 70% of the control samples in the case of E3 gel treatment. The solution of the gel’s main components did not cause any significant decrease in cell viability, while TX-100 caused complete cellular damage (*p* < 0.0001). Figure 5B shows the results of the LDH test. Samples were collected after treatment from the apical and basal compartments of the inserts, and the LDH activity was determined from the solutions. The treatment with E3 gel and E3-HPBCD solution caused non-significant LDH release from the reconstructed human vaginal epithelium compared to the control, but in accordance with MTT results, TX-100 caused significant LDH release from the cells. The TEER values were measured at the end of the biocompatibility test, and the E3-HPBCD solution did not cause significant alterations in TEER values compared to the untreated control (*p* > 0.05) (Table 1). TEER was not measured on inserts treated with E3 gel.

#### 2.1.5. Permeability of E3 Through the Reconstructed Human Vaginal Epithelium

**Figure 6:**
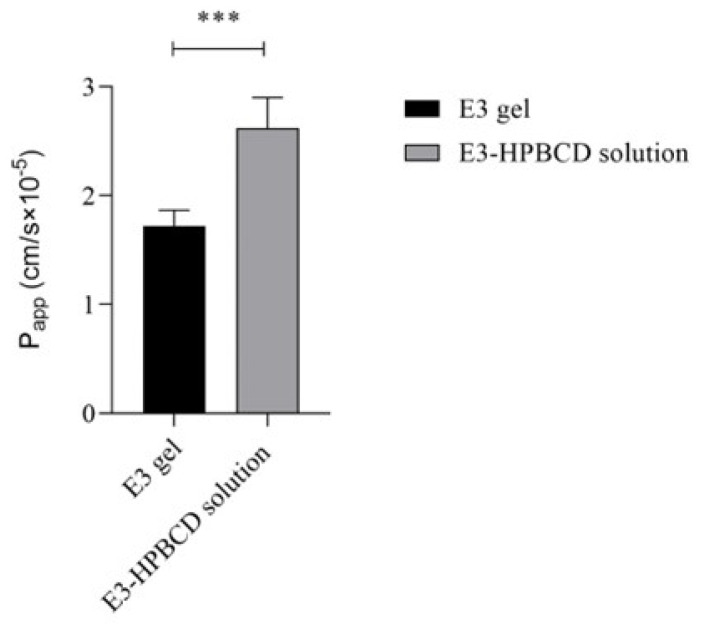
Permeability of E3 through the reconstructed human vaginal epithelium. E3 gel and E3-HPBCD solution were applied on the apical side of the inserts, and the permeability of E3 was determined after two hours of incubation. Values were expressed as mean ± S.D., n = 4–5. The apparent permeability of E3 was significantly higher from the E3-HPBCD solution compared to the E3 gel. *** represents *p* < 0.001.

The permeability of E3 through the human vaginal epithelium was also determined using the E3 gel and E3-HPBCD solutions. E3 quickly permeated the epithelial cell layer grown on porous membranes from both types of samples, indicating that E3 is freely available from the vaginal gel formulation. The permeability of E3 was significantly higher (*p* < 0.001) from the E3-HPBCD solution than from the E3 gel, which is in accordance with the viscous property of the gel (Figure 6).

### 2.2. Discussion

GSM is a chronic, progressive condition characterized by a range of genital, sexual, and urinary symptoms. Early recognition and appropriate management, based on patient-reported symptoms and goals, are essential for maintaining urogenital health [28]. Various vaginal products, including different formulations such as tablets, rings, capsules, pessaries, creams, gels, and ovules, as well as different molecules like E2, E3, promestriene, conjugated equine estrogens, and estrone, have been used, yielding comparable therapeutic outcomes [29].

To our knowledge, the formulation investigated in this study is the first vaginal product to contain both HEC and HPBCD. A recent review of CD-containing pharmaceutical products on the market identified only four vaginal formulations using β-CD. In addition, no vaginal formulations containing other forms of CDs were documented. While 30.1% of existing CD formulations contain HPBCD to date, none of these are specifically designed for vaginal use [25].

Based on the available scientific literature, hydrogels are considered preferable to creams for vaginal drug delivery, as their mucoadhesive properties allow for prolonged retention of the active substance at the site of action. They provide controlled, sustained drug release, reducing the need for frequent applications. Hydrogels are more comfortable as they offer a non-greasy, light texture. Their longer duration of action improves patient adherence compared to creams [30,31].

HEC-based gels offer superior biocompatibility and a lower risk of irritation [32], making them particularly suitable for sensitive vaginal applications. To our knowledge, there is currently only one vaginal gel containing E3, called Blissel (Consilient Health Ltd., Dublin, Ireland). However, this formulation uses polycarbophil and carbomer to make E3 bioavailable [33]. Although these materials can also be used, we believe that our HEC- and HPBCD-based gels are more suitable for vaginal use. As a natural derivative of cellulose, HEC shows greater physiological compatibility than synthetic polymers such as carbomer and polycarbophil [34,35]. In addition, these gels offer more controlled and sustained drug delivery, allowing for a gradual release of active ingredients. The hydrophilic properties of HEC contribute to superior moisture retention, promoting optimal hydration and comfort in the vaginal environment. In addition, HEC-based gels are less likely to disrupt the vaginal pH balance, minimizing the risk of infection or irritation [36].

Its high water solubility characterizes HPBCD, significantly improving poorly water-soluble drugs’ solubility [37]. Its low toxicity makes it a safer option for long-term pharmaceutical applications compared to other CDs. In addition, HPBCD exhibits superior complexation efficiency, improving drug bioavailability and stability while effectively masking unwanted taste or odor. Importantly, HPBCD offers improved biocompatibility, minimizing potential adverse effects [23].

In the cytotoxicity evaluations of the vaginal hydrogel containing HEC and HPBCD as excipients and E3 as the drug, no cytotoxicity observed. According to ISO 10993-5, which addresses the biological evaluation of medical devices and specifically tests for in vitro cytotoxicity, a material is considered to have cytotoxic potential if the viability drops below 70% of the control value [38]. MTT assays performed on HaCaT cells showed a statistically significant increase in cell viability with hydrogel treatment, as evidenced by the increased formation of formazan dye over time compared to the untreated control (*p* < 0.05) [39,40]. In addition, LDH assays showed no significant increase in cytotoxicity associated with gel treatment, confirming the absence of adverse effects on cell membranes. Different dilutions of the E3-containing gel did not cause LDH release from the cells but reduced LDH release compared to the baseline LDH release measured in control cells. An explanation in the literature is that in estradiol-treated neurons, a similar reduction in LDH release was observed in the lipopolysaccharide (LPS)-induced inflammatory process compared to controls, suggesting reduced cell death [41]. Another study points to the inhibitory effect of estrogens on LDH activity [42,43]. As both MTT- and LDH-assays can be influenced by the E3 RTCA method, this was used to attain further information on cell viability and proliferation. This method applies impedance measurements to monitor the status of cells, and thus the metabolic or enzymatic interactions caused by E3 do not affect the measurement. RTCA further supported the above-mentioned findings, showing improved cell adhesion at a 5-fold dilution of the hydrogel, underscoring its potential to promote healthy cell growth. The literature shows that estrogens increase the efficiency of the mitochondrial electron transport chain, enhancing energy production efficiency [40,41]. In contrast to conventional formulations, the demonstrated lack of cytotoxicity of this hydrogel positions it as a superior alternative for vaginal applications.

The bioavailability of E3 from the hydrogel formulation was assessed using reconstructed vaginal epithelial models. Permeation studies demonstrated that E3 is effectively released from the gel matrix and penetrates the vaginal epithelial barrier, ensuring sustained bioavailability. The release profile showed a more gradual permeation from the hydrogel compared to the E3-HPBCD solution, which is particularly advantageous for achieving a prolonged therapeutic effect in the vaginal environment. Despite a more controlled release, the permeation rate was sufficient to ensure effective delivery of E3. This study has several notable strengths that enhance its scientific and clinical significance. It introduces a pioneering vaginal gel formulation, the first of its kind to combine HEC and the E3-HPBCD complex, representing a novel approach to improve estriol delivery in vaginal therapies. The innovative formulation enhances the solubility and bioavailability of estriol, which is essential for optimal therapeutic outcomes. This study is methodologically sound, using a Franz diffusion cell system to rigorously evaluate the estriol release profile and permeability, ensuring high reliability and reproducibility. The use of reconstructed human vaginal epithelium in the permeability assays provides translational value by closely simulating in vivo conditions, thereby increasing the clinical relevance of the findings. Finally, comprehensive biocompatibility testing, including MTT and LDH assays and real-time cell analysis (RTCA), provides a robust assessment of the gel’s safety and cytotoxicity.

Our study also has several limitations, including the short testing period of 72 h. More prolonged exposure to E3-HPBCD or to the vaginal gel might have caused cytotoxicity. It is difficult to find experimental set-ups in cell cultures that effectively model in vivo cytotoxicity. The gel itself is also difficult to physically test for cytotoxicity in cell cultures. In addition, we used only two types of cells in our current experiments (keratinocytes and vaginal epithelial cells), and not endothelial cells. Vascular damage through endothelial cell disruption may present a different mechanism for toxicity, which was not tested in our experiments. Another limitation of our study is the lack of in vivo testing. We plan to conduct in vivo testing in the next phase of development to assess irritation and allergic reactions.

## 3. Conclusions

The novel vaginal gel formulation combining hydroxyethylcellulose (HEC), hydroxypropyl-beta-cyclodextrin (HPBCD), and estriol represents a promising therapeutic option for the treatment of genitourinary syndrome of menopause (GSM). The hydrogel exhibited a consistent viscosity profile, as indicated by measurements using a RheolabQC rotational rheometer. At a constant shear rate of 30 1/s and 25 °C, the viscosity remained low and stable during a 120 s evaluation period, starting at 8.08 Pa·s and reaching a minimum of 7.19 Pa·s. These results confirm the hydrogel’s suitability for vaginal applications, characterized by ease of application, non-greasiness, and comfort. The viscosity graph is included in the Supplementary Materials. In addition to viscosity, the hydrogel’s formulation includes hydroxyethyl cellulose (HEC), which enhances mucoadhesion and hydration, ensuring prolonged retention at the site of action. The inclusion of HPBCD significantly enhances the solubility and bioavailability of estriol, allowing greater permeability across the vaginal epithelium. The gel demonstrated excellent biocompatibility. This hydrogel offers superior patient comfort compared to traditional creams due to its non-greasy, mucoadhesive properties. We have included a detailed characterization of the hydrogel in the Supplementary Materials. These results strongly suggest that this novel vaginal gel formulation could serve as an effective and well-tolerated alternative for women with GSM, although further studies are essential to assess its long-term safety and efficacy. The innovative use of excipients in this formulation represents a significant advancement in the field of vaginal drug delivery.

## 4. Materials and Methods

### 4.1. Materials

HPBCD was purchased from Cyclolab Ltd. (Budapest, Hungary), E3, sodium chloride, and potassium sorbate were obtained from Merck Ltd. (Budapest, Hungary), zinc sulfate and lactic acid were purchased from Hungaropharma Ltd. (Budapest, Hungary), hydroxyethyl cellulose (HEC; Cellosize QP-30000H, Dow Chemicals Midland, MI, USA), and (3-[4,5-dimethylthiazol-2-yl]-2,5-diphenyltetrazolium bromide) (MTT) was obtained from Merck Ltd. (Budapest, Hungary).

A commercially available zinc-containing vaginal gel (JUVIA vaginal gel; Fempharma Europe, LLC, Budapest, Hungary) was supplemented with 0.5% (*m*/*m*) HPBCD and E3 at a final concentration of 15 µg/mL. The main components of the gel are water, hydroxyethyl cellulose, zinc sulfate heptahydrate at a concentration of 20 µM, and lactic acid to set the pH of the gel to 4.50. Potassium sorbate at the final concentration of 0.1% was used as a microbiological preservative. The ready gel was a transparent hydrogel, which contained E3 in a water-soluble HPBCD complex.

### 4.2. Methods

#### 4.2.1. The Methods of In Vitro Release and Membrane Permeation of E3

A vertical Franz diffusion cell system (Hanson Microette TM Topical and Transdermal Diffusion Cell System) was used to test the release and membrane permeation of E3 from the gel. Gel samples (0.3 g) were placed on cellulose-acetate membranes in the donor chamber, and 30% (*v*/*v*) ethanol was used as an acceptor phase at 37 ± 0.5 °C. Experiments were also carried out on membranes impregnated with isopropyl-myristate (IPM). Samples were taken from the acceptor chamber at 15, 30, 60, 90, and 120 min and replaced with fresh acceptor medium. The permeated amount of E3 was determined by an LC-MS method (see later).

#### 4.2.2. Cell Culture

Human skin keratinocytes (HaCaT, Cell Lines Service [CLS], Heidelberg, Germany) were cultured in DMEM supplemented with 1% nonessential amino acids, 10% heat-inactivated fetal bovine serum (FBS), and 1% penicillin–streptomycin solution. The cells were incubated at 37 °C with 5% CO_2_.

Reconstructed human vaginal epithelium (SkinEthic HVE/S/5) was obtained from EPISKIN (Lyon, France). The vaginal epithelial cells were cultured on membrane inserts (surface area 0.5 cm^2^), placed in the supplied growth medium (SGM, EPISKIN) upon arrival, and maintained at 37 °C in an incubator following the manufacturer’s instructions. A permeability test was performed on the reconstructed vaginal epithelium after 24 h of incubation.

#### 4.2.3. The Methods of Biocompatibility Tests on HaCaT Cells

##### MTT Assay

HaCaT cells were seeded on a flat-bottomed 96-well tissue culture plate at a density of 1 × 10^4^ cells/well and grown in a CO_2_ incubator at 37 °C for four days. The cells were treated with the 5-, 10-, and 100-times dilutions of the E3 gel in a complete cell culture medium for 2, 24, and 72 h. Then, the test solutions were removed, and the cells were further incubated in 0.5 mg/mL (3-(4,5-dimethylthiazol-2-yl)-2,5-diphenyl-2H-tetrazolium bromide) solution (MTT-solution) at 37 °C for 4 h. The formed formazan crystals were dissolved in acidic isopropanol (isopropanol:1.0N hydrochloric acid 25:1), and the MTT absorbance was measured at 570 nm against a 690 nm reference wavelength with a FLUOstar OPTIMA Microplate Reader (BMG LABTECH, Offenburg, Germany). Cell viability was expressed as the percentage of the untreated control. We used 2% Triton X-100 (TX-100) as a positive control in the experiments.

##### Lactate Dehydrogenase (LDH) Assay

Cells were treated as described in the MTT test; however, the samples were diluted with 1% FBS-containing medium to avoid a high background. After treatment, supernatants were removed from the cells, and LDH activity was determined using the CyQUANT LDH Cytotoxicity Assay (Invitrogen) according to the manufacturer’s instructions. Cytotoxicity was calculated as follows:

$$Cytotoxicity\;\%=\frac{Compound-treated\;LDH\;activity-Spontaneous\;LDH\;activity}{Maximum\;LDH\;activity-Spontaneous\;LDH\;activity}\times 100$$

##### Real-Time Cell Analysis (RTCA)

HaCaT cells were seeded on 16-well e-plates at a density of 1×104 cells/well, placed in the RTCA DP Instrument (XCelligence system, ACEA Biosciences Inc., San Diego, CA, USA), and incubated in a CO_2_ incubator at 37 °C for 24 h. Cells were treated with the test solutions described in the MTT test, and further incubated for 72 h; the cell index was registered every 25 min. The normalized cell index was calculated by the RTCA Software 2.0 and depicted as a function of time.

##### Biocompatibility Tests on Reconstructed Human Vaginal Epithelium

Inserts containing the reconstructed human vaginal epithelium were treated with E3 gel or with a solution containing the components of the gel, except the gelling agent HEC (E3-HPBCD solution). The components were dissolved in a maintenance medium (SMM, EPISKIN). Treatments were run for 2 h at 37 °C, and the MTT and LDH tests were performed as described above with the following adaptations to the inserts. In the case of E3-gel-treated inserts, the samples were collected for LDH tests only from the basal side due to inaccurate sampling from the viscous-gel-containing apical side. In the case of E3-HPBCD solution-treated inserts, samples were collected both from the apical and basal sides for the LDH test. Finally, the inserts were placed in 0.5 mg/mL MTT solution and further incubated for 24 h at 37 °C. The formazan crystals were dissolved in acidic isopropanol and measured as described earlier. We used 2% TX-100 as a positive control.

#### 4.2.4. Permeability Test of E3 Through the Reconstructed Human Vaginal Epithelium

E3 gel and E3-HPBCD solution were placed on reconstructed human vaginal epithelium grown on porous membrane inserts and incubated at 37 °C. The basal chamber contained FBS. At 60 and 120 min, 1 mL samples were taken from the basal side, and 3 mL acetonitrile was added to the samples to precipitate serum proteins. Samples were centrifuged at 4000 rpm for 6 min, and the supernatants were placed in clear tubes and dried under N2 stream at 50 °C. The permeated amount of E3 was determined by the LC-MS method after derivatization, as described below. The apparent permeability of E3 was calculated with the following formula:

Papp = dQ/dt(1/C0A)

where Papp is the apparent permeability coefficient (cm/s);

dQ/dt is the permeability rate of substances (mol/s);

C0 is the initial concentration of the substances in the apical chamber (mol/mL);

A is the surface area of the membrane (cm^2^).

The integrity of the reconstructed human vaginal epithelium was also tested by the measurement of transepithelial electric resistance (TEER) using a Millicell–ERS voltohmmeter (Merck Inc., Rahway, NJ, USA) and expressed as Ωcm^2^.

#### 4.2.5. LC-MS Method

##### Sample Preparation

Derivatization with pyridine-3-sulfonyl chloride (PSCl) was carried out as follows: To the dried sample or working standards or samples, we added 75 μL of aq. sodium bicarbonate (100 mM, pH 9) and 75 μL of pyridine-3-sulfonyl chloride (3 mg/mL in acetone). The mixture was capped and incubated at 65 °C for 15 min. After cooling, the reaction mixture was transferred to HPLC inserts and subjected to LC/MS analysis.

Next, 2 µL of sample solution was injected into the LC/MS. The experiment was performed in three replicates for each time point. Determination was performed on a Kinetex Polar C18 (100 × 3.0 mm, 2,6 µm, 100 Å) column, using an Accela HPLC system (Thermo Electron Corp., San Jose, CA, USA) eluted with a gradient of acetonitrile (A) and water (B) containing 0.1% (*v*/*v*) formic acid each. The gradient was from 60% A (hold for 2 min) to 90% A over 7 min, hold for 6 min, return to initial conditions, and hold for 5 min to equilibrate the column. The LC system was interfaced with a Thermo LTQ XL mass spectrometer (Thermo Electron Corp., San Jose, CA, USA) and operated in both positive and negative-ion ESI modes for ionization. The ion injection time was 100 ms. The ESI parameters were as follows: spray voltage of 5 kV, source heater temperature at 280 °C, capillary temperature at 300 °C, sheath gas flow at 25 units (N2), and auxiliary gas flow at 8 units (N2). The tray temperature was maintained at 12 °C, while the column oven was set to 30 °C to ensure optimal retention of the compounds in the reaction mixtures.MS2 product-ion scans were acquired following collision-induced dissociation, using helium as the target gas. Compound identification was performed based on their retention times (tR), HESI mass spectra, and MS2 data, with authentic compounds serving as references. Recovery was calculated to be above 90% for all analytes of interest. E3 hormones were determined as their PSC derivative (pyridine-3-sulfonyl derivative) [44].

#### 4.2.6. Statistical Analysis

The data were analyzed with GraphPad Prism version 9.1.2 (GraphPad Software, San Diego, CA, USA) and are presented as means ± SD. Comparisons between two groups were made using an unpaired *t*-test, while comparisons involving more than two groups were conducted using analysis of variance. A *p*-value of less than 0.05 was considered statistically significant.

## Figures and Tables

**Table 1:** Transepithelial electric resistance (TEER) of reconstructed human vaginal epithelium after biocompatibility test.

	TEER Value (Ωcm^2^)
E3-HPBCD solution (n = 3)	46.5 ± 3.46
TX-100 (n = 3)	32.5 ± 1
Control (n = 3)	44.33 ± 4.01

## Data Availability

The original contributions presented in this study are included in the article/Supplementary Materials. Further inquiries can be directed to the corresponding author.

## Supplementary Materials

The following supporting information can be downloaded at: https://www.mdpi.com/article/10.3390/gels10120823/s1, Figure S1: Viscosity was measured by a RheolabQC rotational rheometer (Anton Paar, Graz, Austria) equipped with a measuring cylinder CC27; applying constant shear rate at 30 1/s, at 25 °C. Viscosity of the gel was low and remained almost constant during the 120 s of investigation, starting at 8.08 Pa·s, and reaching the minimum of 7.19 Pa·s by the end of the test.
