# Supplementary material for: Development and Bioavailability Assessment of an Estriol-Containing Vaginal Hydrogel
Source: Gels. 2024 Dec 13;10(12):823. doi: 10.3390/gels10120823 (PMC11675891; doi:10.3390/gels10120823)
Supplement: Supplementary file 1 [file gels-10-00823-s001.zip › gels-3341937-supplementary.pdf]

gels

MDPI

Supplementary Materials

Viscosity curve of E3-HPBCD gel

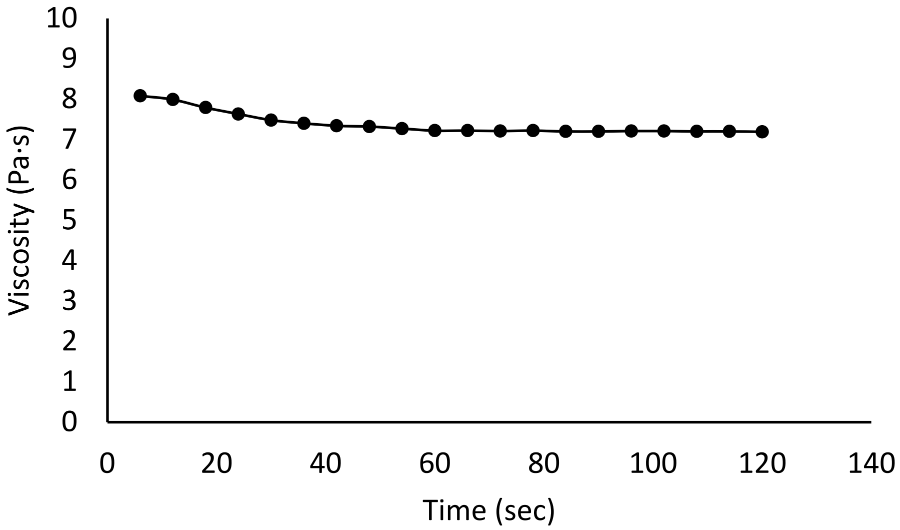

Figure S1. Viscosity was measured by a RheolabQC rotational rheometer (Anton Paar, Graz, Austria) equipped with a measuring cylinder CC27; applying constant shear rate at 30 1/s, at 25 °C. Viscosity of the gel was low and remained almost constant during the 120 sec of investigation, starting at 8.08 Pa·s, and reaching the minimum of 7.19 Pa·s by the end of the test.
